# Prognostic and Treatment-Context Associations of EASIX and IPI in Extensive-Stage Small-Cell Lung Cancer Patients Receiving First-Line Therapy

**DOI:** 10.3390/cancers18172888

**Published:** 2026-09-06

**Authors:** Hatice Ayyıldız Sevim, Galip Can Uyar, Güner Akgüner, Hayriye Şahinli

**Affiliations:** Department of Medical Oncology, Ankara Etlik City Hospital, Ankara 06170, Türkiye; g.can_uyar@hotmail.com (G.C.U.); gunerakguner@gmail.com (G.A.); dr.hayriye@hotmail.com (H.Ş.)

**Keywords:** extensive-stage small-cell lung cancer, Endothelial Activation and Stress Index, Inflammatory Prognostic Index, overall survival, prognostic biomarkers

## Abstract

Background: The comparative prognostic value of the Endothelial Activation and Stress Index (EASIX) and the Inflammatory Prognostic Index (IPI) in extensive-stage small-cell lung cancer remains unclear. This study aimed to compare the prognostic value of EASIX and IPI and assess their contribution beyond established clinical factors. Methods: This retrospective single-center study included 129 patients with de novo extensive-stage small-cell lung cancer who initiated first-line systemic therapy between December 2022 and October 2025. Pretreatment EASIX and IPI values were analyzed as log_2_-transformed continuous variables in multivariable Cox models. Overall survival was the primary outcome, and first-line progression-free survival was the secondary outcome. Results: During a median follow-up of 26.91 months, 116 deaths and 123 progression-or-death events occurred. Each doubling of the EASIX value was independently associated with a higher hazard of death (adjusted hazard ratio, 1.14; 95% confidence interval, 1.01–1.28; *p* = 0.019), whereas IPI was not independently associated with overall survival. Neither index was independently associated with first-line progression-free survival. Adding EASIX to the clinical model improved model fit and increased the C-index from 0.619 to 0.650, whereas IPI provided no additional prognostic contribution. Conclusions: EASIX may provide prognostic information beyond established clinical factors for overall survival in extensive-stage small-cell lung cancer. These findings warrant validation in larger multicenter cohorts.

## Simple Summary

Patients with extensive-stage small-cell lung cancer can have substantially different outcomes, highlighting the need for simple and readily available prognostic markers. In this study, we compared two blood test-based indices, the Endothelial Activation and Stress Index (EASIX) and the Inflammatory Prognostic Index (IPI), in 129 patients receiving first-line systemic therapy. Higher EASIX values were associated with shorter overall survival in the primary analyses, and adding EASIX to established clinical factors provided a modest improvement in prognostic information. In contrast, IPI did not provide independent prognostic information. Overall, EASIX may represent a practical prognostic marker in extensive-stage small-cell lung cancer, but its clinical value requires confirmation in larger, independent multicenter studies.

## 1. Introduction

Small cell lung cancer (SCLC) is an aggressive neuroendocrine malignancy that accounts for approximately 15% of all lung cancer cases and is closely associated with tobacco exposure [1]. Owing to its rapid tumor growth and propensity for early metastatic dissemination, the majority of patients present with extensive-stage disease at diagnosis [2]. Although SCLC is initially sensitive to platinum-based chemotherapy, durable disease control is rarely achieved. Therefore, outcomes in extensive-stage SCLC remain poor; median overall survival is generally below 12 months, and prolonged survival is achieved in only a small minority of patients [3].

Although knowledge of the molecular and biological landscape of SCLC has expanded, these findings have not yet assumed a definitive role in routine diagnostic practice. Nevertheless, multiple biological mechanisms are thought to underlie the rapidly progressive clinical course and poor prognosis of the disease. In particular, systemic inflammation, cancer cachexia, and sarcopenia have been reported as factors associated with unfavorable survival outcomes in patients with SCLC [4,5,6]. Moreover, mechanisms facilitating metastatic spread, intratumoral heterogeneity, and angiogenesis are considered important biological processes that may contribute to disease progression and treatment resistance [7,8,9]. One study detected vasculogenic mimicry in SCLC tumor specimens. This process is characterized by the formation of vascular-like structures by tumor cells exhibiting endothelial-like features. This phenomenon was suggested to be associated with more aggressive tumor behavior, reduced sensitivity to cisplatin, and poorer overall survival (OS) [10]. Another clinical study found that baseline levels of circulating endothelial cells were increased in patients with SCLC compared with healthy controls. This increase, considered a marker of vascular injury, was associated with a lower treatment response rate and poorer progression-free survival (PFS) [11].

The Endothelial Activation and Stress Index (EASIX) integrates LDH, serum creatinine, and platelet count into a single laboratory-based measure. It was originally developed to estimate the risk of graft-versus-host disease-related complications after allogeneic stem-cell transplantation and was subsequently investigated as a prognostic marker in several hematological malignancies [12,13,14].

More recent studies have extended the evaluation of EASIX beyond hematological disorders. In solid tumors, studies involving SCLC, urothelial cancer, and metastatic pancreatic cancer have reported associations between EASIX and clinical outcomes [15,16,17]. Evidence evaluating the prognostic relevance of EASIX in SCLC remains limited and is derived primarily from a single study [15]. Considering the systemic burden, rapidly progressive biology, and poor prognosis of this disease, EASIX may represent a practical and informative biomarker to support risk stratification.

EASIX may indirectly reflect endothelial injury and systemic stress responses, both of which play important roles in cancer progression, metastatic dissemination, and the development of treatment resistance. Among the components of the score, elevated LDH levels may be associated with hypoxic tumor metabolic activity and rapid tumor growth, whereas increased creatinine levels may indicate systemic stress or organ dysfunction. Changes in platelet count may reflect inflammatory response, coagulation activation, or impaired bone marrow reserve. Therefore, EASIX may be considered a practical and integrated marker that captures not only tumor biology but also the systemic host response to the disease [16].

The Inflammatory Prognostic Index (IPI) is a composite inflammatory marker incorporating C-reactive protein (CRP), the neutrophil-to-lymphocyte ratio (NLR), and serum albumin and is calculated as CRP × NLR/serum albumin. Initially developed for non-small-cell lung cancer, IPI has been reported to reflect both systemic inflammation and nutritional status, with higher values being associated with poorer survival outcomes [18]. A recently published study demonstrated that higher IPI values were associated with shorter OS in patients with extensive-stage SCLC and that this association remained significant in multivariable analysis [19]. Nevertheless, evidence regarding IPI in extensive-stage SCLC remains limited. To our knowledge, no previous study has directly compared EASIX and IPI within the same cohort of patients with extensive-stage SCLC. Therefore, evaluating these two indices together may contribute to a better understanding of the prognostic relevance of endothelial stress and inflammation- and nutrition-related processes.

Because EASIX and IPI can be calculated using readily available and low-cost laboratory parameters, they may represent practical tools for clinical assessment. In this study, we aimed to comparatively evaluate the associations of pretreatment EASIX and IPI values with OS and PFS1 in patients with extensive-stage SCLC receiving first-line systemic therapy.

## 2. Materials and Methods

### 2.1. Patient Selection and Data Collection

This retrospective, single-center study was conducted using data from patients followed at the Department of Medical Oncology, Ankara Etlik City Hospital. Consecutive patients with de novo extensive-stage small-cell lung cancer who initiated first-line systemic therapy between December 2022 and October 2025 were screened. The data cutoff was 30 June 2026. Ethical approval for the study was obtained from the Scientific Research Evaluation and Ethics Committee of Ankara Etlik City Hospital (Decision No.: AEŞH-BADEK1-2026-414; 6 May 2026), and the study was conducted in accordance with the Declaration of Helsinki. The requirement for informed consent was waived due to the retrospective design of the study and the use of anonymized data.

The inclusion criteria were as follows: age ≥ 18 years, histologically confirmed de novo extensive-stage small-cell lung cancer, receipt of first-line systemic therapy, and availability of clinical, laboratory, and survival data. The exclusion criteria were defined as the presence of a concurrent active second malignancy, limited-stage disease, and missing required clinical or laboratory data. Clinical records and laboratory databases were retrospectively reviewed.

### 2.2. Data Variables

Collected variables included demographic and clinical characteristics, including age, sex, body mass index, smoking status and exposure, Eastern Cooperative Oncology Group (ECOG) performance status, and the presence of brain, liver, bone, pleural, adrenal, and lymph node metastases. First-line treatment characteristics included the platinum backbone (cisplatin–etoposide or carboplatin–etoposide) and atezolizumab exposure. First-line platinum backbone (cisplatin or carboplatin) was selected by the treating physician according to routine clinical practice, taking into account patient characteristics, renal function, comorbidities, and performance status. Atezolizumab was administered to eligible patients according to routine institutional practice. During most of the study period, routine access to atezolizumab for first-line extensive-stage SCLC was limited because national reimbursement for this indication was not yet available in Türkiye. Reimbursement became only available toward the end of the study period; therefore, atezolizumab exposure in this cohort reflects not only clinical treatment selection but also temporal differences in treatment access. Laboratory parameters included hemoglobin; neutrophil, lymphocyte, and platelet counts; as well as lactate dehydrogenase (LDH), serum creatinine, C-reactive protein (CRP), and serum albumin levels. Baseline laboratory parameters were defined as the closest available measurements obtained within 7 days before initiation of first-line systemic therapy. The neutrophil-to-lymphocyte ratio (NLR) was calculated by dividing the absolute neutrophil count by the absolute lymphocyte count.

EASIX was calculated using the standard formula: EASIX = LDH (U/L) × serum creatinine (mg/dL)/platelet count (×10^9^/L). In the institutional laboratory reporting system, platelet counts were recorded as ×10^3^/µL, which is numerically equivalent to ×10^9^/L; therefore, platelet values were used without numerical conversion. IPI was calculated as CRP (mg/L) × NLR/serum albumin (g/L).

Initial staging included PET/CT or thoracoabdominopelvic (TAP) CT, depending on the clinical context, together with brain MRI. The first response assessment during first-line treatment was performed after three cycles of chemotherapy. Subsequent imaging assessments were generally performed at approximately 3–4-month intervals or earlier when clinically indicated. Follow-up imaging consisted of PET/CT or TAP CT depending on the clinical context, with PET/CT used more commonly. Radiographic response and progression were assessed according to RECIST criteria.

### 2.3. Study Endpoints and Statistical Analysis

All analyses were based on the complete eligible cohort. Because the study was retrospective and included all consecutive patients meeting the prespecified eligibility criteria during the study period, no formal a priori sample-size calculation was performed. No missing values were present for the variables included in the primary analyses; therefore, no missing-data imputation was required.

Continuous variables were summarized as medians with interquartile ranges (IQRs), and categorical variables were summarized as counts and percentages. Baseline characteristics were presented for the overall cohort and according to first-line atezolizumab exposure. Between-group differences were quantified using absolute standardized mean differences (SMDs) rather than hypothesis-testing *p* values. For continuous variables, SMDs were calculated as the difference in group means divided by the pooled standard deviation; for binary variables, the difference in proportions was standardized using the pooled variance of the proportions. Absolute SMD values < 0.10 were considered to indicate negligible imbalance, with progressively larger values reflecting greater baseline imbalance.

The Endothelial Activation and Stress Index (EASIX) was recalculated as lactate dehydrogenase (U/L) × serum creatinine (mg/dL)/platelet count (×10^9^/L). The Inflammatory Prognostic Index (IPI) was calculated as C-reactive protein (mg/L) × neutrophil-to-lymphocyte ratio/serum albumin (g/L), with the neutrophil-to-lymphocyte ratio calculated as the absolute neutrophil count divided by the absolute lymphocyte count. Because both indices had markedly right-skewed distributions, they were log_2_-transformed before regression modeling. Consequently, the hazard ratios (HRs) for EASIX and IPI represent the relative change in the hazard of the relevant outcome associated with each doubling of the respective index values. Their distributions before and after transformation were examined using histograms and kernel density estimates.

Overall survival (OS) was calculated from initiation of first-line systemic therapy to death from any cause; patients who were alive at the data cutoff were censored at the date of last follow-up. First-line progression-free survival (PFS1) was calculated from initiation of first-line systemic therapy to radiographic or clinical disease progression or death from any cause, whichever occurred first. Clinical progression was defined as clinically meaningful symptomatic or functional deterioration attributed to disease progression by the treating oncologist and leading to a change or discontinuation of first-line treatment. Patients without an event were censored at the date of the last adequate disease assessment. Median follow-up was estimated using the reverse Kaplan–Meier method. Median OS and PFS1 were estimated using the Kaplan–Meier product-limit estimator, with confidence intervals obtained by inversion of pointwise complementary log–log-transformed confidence bands. Time-specific survival probabilities at 6 and 12 months for PFS1 and at 12, 18, and 24 months for OS were estimated using Greenwood variance with complementary log–log transformation. Survival estimates according to first-line atezolizumab exposure were unadjusted and descriptive; no formal between-group hypothesis test was performed because treatment allocation was nonrandomized and the estimates were not intended to represent causal treatment effects.

Univariable Cox proportional hazards models were used to examine associations of demographic characteristics, clinical factors, metastatic sites, treatment characteristics, baseline laboratory variables, EASIX, and IPI with OS and PFS1. The primary multivariable Cox models included covariates selected a priori on the basis of clinical relevance and the study objectives, without stepwise selection or selection according to univariable statistical significance. The prespecified adjustment set comprised age, sex, Eastern Cooperative Oncology Group (ECOG) performance status, body mass index (BMI), liver metastasis, brain metastasis, bone metastasis, platinum backbone, first-line atezolizumab exposure, log_2_-transformed EASIX, and log_2_-transformed IPI. Age and BMI were entered continuously, with HRs expressed per 10-year and per 5-kg/m^2^ increase, respectively. ECOG performance status was modeled as 2–3 versus 0–1; platinum backbone as carboplatin–etoposide versus cisplatin–etoposide; and metastatic sites and atezolizumab exposure as binary variables. EASIX and IPI were entered simultaneously in the primary models to estimate their mutually adjusted associations. Palliative radiotherapy was not included as a baseline covariate because it could have been administered after treatment initiation, introducing potential time-dependent and post-baseline biases. Tied event times were handled using the Efron method. Results are reported as HRs with Wald 95% confidence intervals (CIs).

The proportional hazards assumption was assessed using global and covariate-specific Schoenfeld residual-based tests. When evidence of non-proportional hazards was detected, targeted models incorporating time-varying coefficients and additional sensitivity analyses were performed. Specifically, the targeted PFS1 model incorporated a log_2_-EASIX × log(time/6) interaction term, with time expressed in months. In this parameterization, the EASIX main-effect HR represents its association at 6 months, whereas the interaction ratio describes the change in the EASIX log-HR over logarithmic follow-up time.

Potential nonlinear associations of EASIX and IPI with OS and PFS1 were evaluated using restricted cubic spline models. Three knots were placed at the 10th, 50th, and 90th percentiles of the corresponding log_2_-transformed index distribution. The median value was used as the reference and assigned an HR of 1.0. EASIX spline models were adjusted for the complete prespecified clinical covariate set and log_2_-IPI, whereas IPI spline models were adjusted for the same clinical covariates and log_2_-EASIX. Overall associations were evaluated using joint Wald tests of all spline terms, and nonlinearity was assessed using Wald tests of the nonlinear spline component.

To evaluate the comparative and incremental prognostic contribution of EASIX and IPI, four prespecified nested models were fitted separately for OS and PFS1: a clinical model containing the nine prespecified clinical covariates; the clinical model plus log_2_-EASIX; the clinical model plus log_2_-IPI; and the clinical model containing both log_2_-EASIX and log_2_-IPI. Relative model fit was evaluated using log-likelihood values and the Akaike information criterion (AIC), with lower AIC values indicating better relative fit. Nested models were compared using likelihood-ratio tests. Discrimination was summarized using Harrell’s concordance index, with percentile 95% CIs obtained from 1000 patient-level nonparametric bootstrap resamples using a fixed random seed. These analyses were intended to assess comparative and incremental prognostic information and not to develop or validate a clinical prediction model.

Several prespecified sensitivity analyses were conducted. First, associations were expressed per IQR and per 1-standard-deviation increase in the log_2_-transformed indices; these represented alternative scalings of the same model coefficients and therefore did not alter model fit or statistical significance. Second, the original EASIX and IPI distributions were winsorized at the 1st and 99th percentiles before log_2_ transformation. Third, influence-restricted analyses excluded observations for which the absolute leave-one-out DFBETAS value exceeded 2/√*n* for the relevant biomarker and endpoint; these exclusions were used only for sensitivity assessment and did not modify the primary cohort. Fourth, models were additionally adjusted for calendar year of treatment initiation, entered continuously. Fifth, individual-level Huber–White sandwich standard errors were calculated while retaining the original model coefficients. Sixth, the separate indicators for liver, brain, and bone metastases were replaced by the total number of documented baseline metastatic sites across the brain, liver, bone, pleura, adrenal glands, and lymph nodes.

Treatment-restricted sensitivity analyses were also performed. Among patients who received first-line atezolizumab, EASIX and IPI were evaluated in separate parsimonious Cox models adjusted for age, ECOG performance status, liver metastasis, brain metastasis, and platinum backbone. Within the cisplatin–etoposide and carboplatin–etoposide subgroups, biomarker-specific models were adjusted for age, ECOG performance status, liver metastasis, and first-line atezolizumab exposure. These restricted analyses were considered exploratory because of their smaller sample sizes and event counts.

Potential treatment-context heterogeneity was evaluated using formal interaction terms. Separate marker-specific models tested log_2_-EASIX × atezolizumab, log_2_-IPI × atezolizumab, log_2_-EASIX × platinum backbone, and log_2_-IPI × platinum backbone interactions for both OS and PFS1. Interaction models included the full clinical adjustment set, the relevant treatment exposure, the relevant biomarker main effect, and the corresponding interaction term. Group-specific biomarker HRs were obtained from the appropriate linear combinations of the main-effect and interaction coefficients. These analyses were considered exploratory treatment-effect modification analyses and were not interpreted as validation of a predictive biomarker.

Pairwise associations among EASIX, IPI, their constituent laboratory variables, hemoglobin, and BMI were summarized using Spearman rank-correlation coefficients. The lower triangular correlation matrix displayed Spearman’s ρ values rounded to two decimal places. Because Spearman correlation is invariant to monotonic transformations, identical coefficients would be obtained using the original or log_2_-transformed indices. Correlations between each composite index and its constituent variables were interpreted as partly structural. The correlation matrix was descriptive; individual correlation-test *p* values and significance symbols were not displayed, and no inferential conclusions were based on individual pairwise correlations.

All tests were two-sided, and *p* < 0.05 was considered statistically significant. No adjustment for multiple comparisons was applied to the exploratory sensitivity or interaction analyses; these findings were therefore interpreted as hypothesis-generating. No biomarker cutoff or categorization was used, and no receiver operating characteristic, Youden index, or Kaplan–Meier analysis based on biomarker-defined groups was performed.

All statistical analyses were performed using Python version 3.12. Data processing and numerical operations were conducted using pandas version 2.2.3 and NumPy version 2.2.6; statistical functions were implemented using SciPy version 1.15.3; Cox proportional hazards models, covariance estimates, and related regression procedures were performed using statsmodels version 0.14.4; and graphical outputs were generated using Matplotlib version 3.10.3 and seaborn version 0.13.2. A fixed random seed was used for bootstrap-based analyses to ensure reproducibility.

## 3. Results

Following the application of the eligibility criteria, a total of 129 patients with de novo extensive-stage SCLC who received first-line systemic therapy were included in the final analysis. Of these, 77 patients (59.7%) received platinum–etoposide chemotherapy alone, whereas 52 (40.3%) received platinum–etoposide plus atezolizumab. The median age was 64 years (IQR, 59–69), and 106 patients (82.2%) were male. Overall, 86.0% of the patients were current or former smokers, and 76.7% had an ECOG performance status of 0–1. The most frequent metastatic sites were the lymph nodes (65.1%), bone (49.6%), liver (34.1%), and brain (24.0%). First-line treatment consisted of cisplatin–etoposide in 60 patients (46.5%) and carboplatin–etoposide in 69 patients (53.5%). The median baseline EASIX and IPI values were 0.85 (IQR, 0.55–1.49) and 2.18 (IQR, 0.83–7.71), respectively. Table 1 summarizes the baseline demographic, clinical, disease-related, treatment, and laboratory characteristics of the cohort.

EASIX and IPI showed right-skewed distributions in their original scales, whereas the skewness of both indices was reduced after log_2_ transformation (Supplementary Figure S1).

In the Spearman correlation analysis, EASIX was strongly positively correlated with LDH (ρ = 0.79), moderately inversely correlated with platelet count (ρ = −0.51), and weakly to moderately positively correlated with creatinine (ρ = 0.34). IPI was strongly positively correlated with CRP (ρ = 0.89), moderately to strongly positively correlated with NLR (ρ = 0.63), and moderately inversely correlated with albumin (ρ = −0.45). The correlation between EASIX and IPI was weak (ρ = 0.19) (Supplementary Figure S2).

The OS analysis included 129 patients, with 116 deaths. In the univariable analysis, ECOG performance status 2–3, the presence of liver metastasis, and higher EASIX values were associated with an increased risk of death, whereas first-line atezolizumab use was associated with a lower risk of death. The association between IPI and OS did not reach statistical significance (HR, 1.08; 95% CI, 1.00–1.16; *p* = 0.057). In the multivariable analysis, ECOG performance status 2–3 (adjusted HR, 1.98; 95% CI, 1.27–3.10; *p* = 0.003), the presence of liver metastasis (adjusted HR, 1.68; 95% CI, 1.05–2.70; *p* = 0.031), and each doubling of EASIX (adjusted HR, 1.14; 95% CI, 1.01–1.28; *p* = 0.019) were associated with an increased hazard of death. First-line atezolizumab use remained associated with a lower hazard of death (adjusted HR, 0.62; 95% CI, 0.40–0.97; *p* = 0.036). In the multivariable model that simultaneously included EASIX and IPI, no statistically significant association was observed between IPI and OS (adjusted HR, 1.06; 95% CI, 0.97–1.15; *p* = 0.179) (Table 2).

Among the 129 patients evaluated for PFS1, 123 experienced progression or death. In the univariable Cox analysis, ECOG performance status 2–3 and each twofold increase in EASIX were associated with a higher risk of progression or death, whereas first-line atezolizumab exposure was associated with a lower risk. IPI was not significantly associated with PFS1 (HR, 1.04; 95% CI, 0.97–1.12; *p* = 0.234). In the multivariable analysis, ECOG performance status 2–3 was associated with an increased risk of progression or death (adjusted HR, 2.02; 95% CI, 1.28–3.17; *p* = 0.002). First-line atezolizumab use remained associated with a lower risk (adjusted HR, 0.41; 95% CI, 0.26–0.64; *p* < 0.001). In the multivariable model that simultaneously included EASIX and IPI, neither EASIX (adjusted HR, 1.01; 95% CI, 0.88–1.17; *p* = 0.850) nor IPI (adjusted HR, 1.01; 95% CI, 0.93–1.09; *p* = 0.786) was significantly associated with PFS1 (Table 3).

Across the complete set of univariable Cox models, ECOG performance status 2–3, liver or adrenal metastasis, a greater number of metastatic sites, and higher EASIX values were associated with a higher risk of death. Conversely, higher albumin levels and first-line atezolizumab use were associated with a lower risk of death. For PFS1, ECOG performance status 2–3, adrenal metastasis, and higher EASIX values were associated with an increased risk of progression or death, whereas first-line atezolizumab use was associated with a lower risk. IPI was not significantly associated with either OS or PFS1 (Supplementary Table S1).

In the alternative multivariable models, EASIX remained significantly associated with OS when added to the clinical model (adjusted HR, 1.19; 95% CI, 1.05–1.35; *p* = 0.006). The addition of EASIX significantly improved model fit, with the AIC decreasing from 925.28 to 919.78 and the Harrell C-index increasing from 0.619 to 0.650 (likelihood-ratio test *p* = 0.006). In contrast, adding IPI alone to the clinical model did not significantly improve model fit (*p* = 0.122). The model incorporating both indices showed a significant improvement over the clinical model alone (*p* = 0.013). However, adding IPI to the EASIX-containing model provided no further improvement (*p* = 0.277), whereas adding EASIX to the IPI-containing model significantly improved model fit (*p* = 0.012) (Supplementary Table S2).

In the alternative multivariable models for PFS1, neither EASIX (adjusted HR, 1.02; 95% CI, 0.89–1.17; *p* = 0.817) nor IPI (adjusted HR, 1.01; 95% CI, 0.94–1.09; *p* = 0.763) was significantly associated with PFS1 when added separately to the clinical model. The addition of EASIX, IPI, or both indices did not improve model fit compared with the clinical model, with likelihood-ratio test *p* values of 0.818, 0.763, and 0.939, respectively. Harrell’s C-index also remained essentially unchanged across the models. In all models, ECOG performance status 2–3 was associated with an increased risk of progression or death, whereas first-line atezolizumab use was associated with a lower risk (Supplementary Table S3).

Across the sensitivity analyses, the association between EASIX and OS was generally maintained when the index was rescaled per IQR or standard deviation, winsorized, analyzed using robust standard errors, or evaluated in the alternative metastatic-burden model. In the influence-restricted analysis, EASIX remained significantly associated with OS and was also significantly associated with PFS1. However, after additional adjustment for treatment-start year, the association between EASIX and OS was attenuated and was no longer statistically significant (adjusted HR, 1.11; 95% CI, 0.96–1.27; *p* = 0.151). IPI was not significantly associated with OS or PFS1 in any of the sensitivity analyses. In platinum-restricted analyses, EASIX was associated with both OS (adjusted HR, 1.49; 95% CI, 1.18–1.89; *p* < 0.001) and PFS1 (adjusted HR, 1.32; 95% CI, 1.06–1.65; *p* = 0.012) among patients receiving carboplatin–etoposide, whereas no significant association was observed in the cisplatin–etoposide subgroup. In the time-varying PFS1 model, neither the EASIX association at 6 months nor its change over time reached statistical significance (Supplementary Table S4).

In the analysis restricted to the 52 patients who received first-line atezolizumab, there were 41 deaths in the OS analysis and 46 progression-or-death events in the PFS1 analysis. Each doubling of EASIX was associated with a higher risk of death and progression or death in both univariable and multivariable analyses. In the multivariable analyses, the adjusted HR for EASIX was 1.63 for OS (95% CI, 1.15–2.30; *p* = 0.006) and 1.54 for PFS1 (95% CI, 1.12–2.12; *p* = 0.008). In contrast, no statistically significant association was observed between IPI and either OS (adjusted HR, 1.05; 95% CI, 0.91–1.22; *p* = 0.490) or PFS1 (adjusted HR, 1.03; 95% CI, 0.89–1.18; *p* = 0.710) (Supplementary Table S5).

In the interaction analyses according to first-line atezolizumab exposure, the associations of EASIX with survival outcomes differed between the treatment groups. Among patients who did not receive atezolizumab, EASIX was not significantly associated with either OS or PFS1. In contrast, among patients receiving atezolizumab, each doubling of the EASIX value was associated with a higher risk of death (HR, 1.55; 95% CI, 1.14–2.12) and a higher risk of progression or death (HR, 1.44; 95% CI, 1.05–1.97). The interaction between EASIX and atezolizumab exposure was statistically significant for both OS (interaction HR, 1.48; 95% CI, 1.07–2.05; *p* = 0.019) and PFS1 (interaction HR, 1.51; 95% CI, 1.08–2.10; *p* = 0.016). Similarly, the associations of EASIX with survival outcomes differed according to platinum backbone. EASIX was not significantly associated with OS or PFS1 in the cisplatin–etoposide group, whereas it was associated with both OS (HR, 1.44; 95% CI, 1.16–1.79) and PFS1 (HR, 1.37; 95% CI, 1.10–1.70) in the carboplatin–etoposide group. The interaction between EASIX and platinum backbone was statistically significant for OS (interaction HR, 1.46; 95% CI, 1.13–1.89; *p* = 0.004) and PFS1 (interaction HR, 1.56; 95% CI, 1.20–2.03; *p* < 0.001). No statistically significant interactions were observed between IPI and either atezolizumab exposure or platinum backbone (Supplementary Table S6).

The median follow-up estimated using the reverse Kaplan–Meier method was 26.91 months (95% CI, 19.75–28.25) in the overall cohort. Median OS was 9.26 months (95% CI, 8.28–10.61), and median PFS1 was 6.64 months (95% CI, 6.05–7.10). The estimated PFS1 rates were 61.2% at 6 months and 10.3% at 12 months, whereas the estimated OS rates at 12, 18, and 24 months were 30.2%, 13.5%, and 5.7%, respectively. In the descriptive treatment-group analyses, the median OS and PFS1 were 8.51 and 6.18 months, respectively, among patients receiving chemotherapy alone, compared with 10.02 and 7.72 months among those receiving chemotherapy plus atezolizumab. Time-specific survival estimates according to treatment group are presented in Supplementary Table S7.

**Figure 1:**
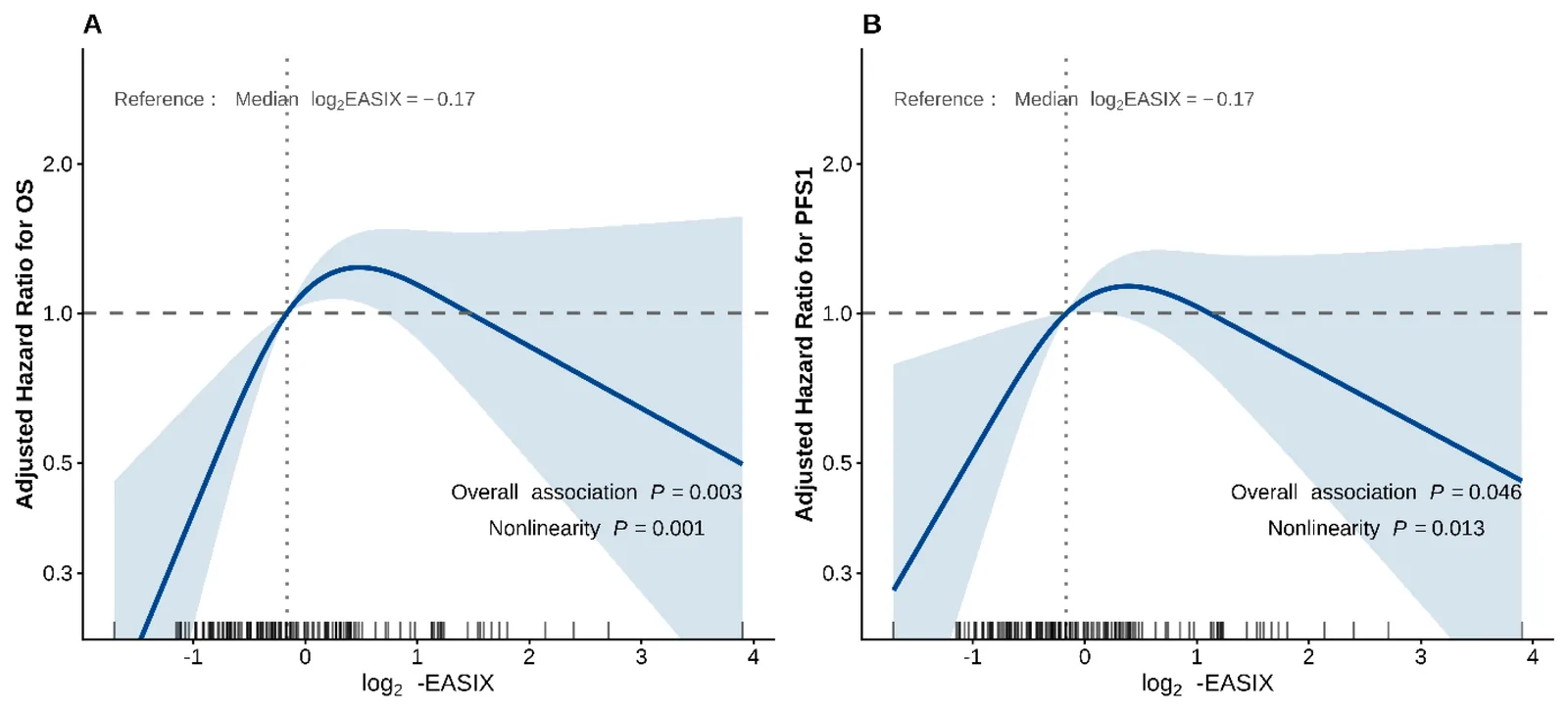
Adjusted continuous associations of baseline EASIX with overall and first-line progression-free survival. (**A**) shows the association between log_2_-transformed EASIX and OS values, and (**B**) shows the association with PFS1. Solid lines represent adjusted HRs, and shaded areas represent 95% CIs. Restricted cubic spline models used three knots at the 10th, 50th, and 90th percentiles, with the median log_2_(EASIX) value (−0.17) as the reference. Models were adjusted for age; sex; ECOG performance status; body mass index; liver, brain, and bone metastases; platinum backbone; first-line atezolizumab exposure; and log_2_-IPI. *p* values for the overall association and nonlinearity were 0.003 and 0.001 for OS and 0.046 and 0.013 for PFS1, respectively. Estimates at the extremes of the distribution should be interpreted cautiously because of the wide confidence intervals. Abbreviations: CI, confidence interval; EASIX, Endothelial Activation and Stress Index; ECOG, Eastern Cooperative Oncology Group; HR, hazard ratio; IPI, Inflammatory Prognostic Index; OS, overall survival; PFS1, first-line progression-free survival.

In the adjusted restricted cubic spline analyses, baseline EASIX showed significant overall and nonlinear associations with OS (*p* for overall association = 0.003; *p* for nonlinearity = 0.001). Significant overall and nonlinear associations were also observed between EASIX and PFS1 (*p* = 0.046 and *p* = 0.013, respectively) (Figure 1).

**Figure 2:**
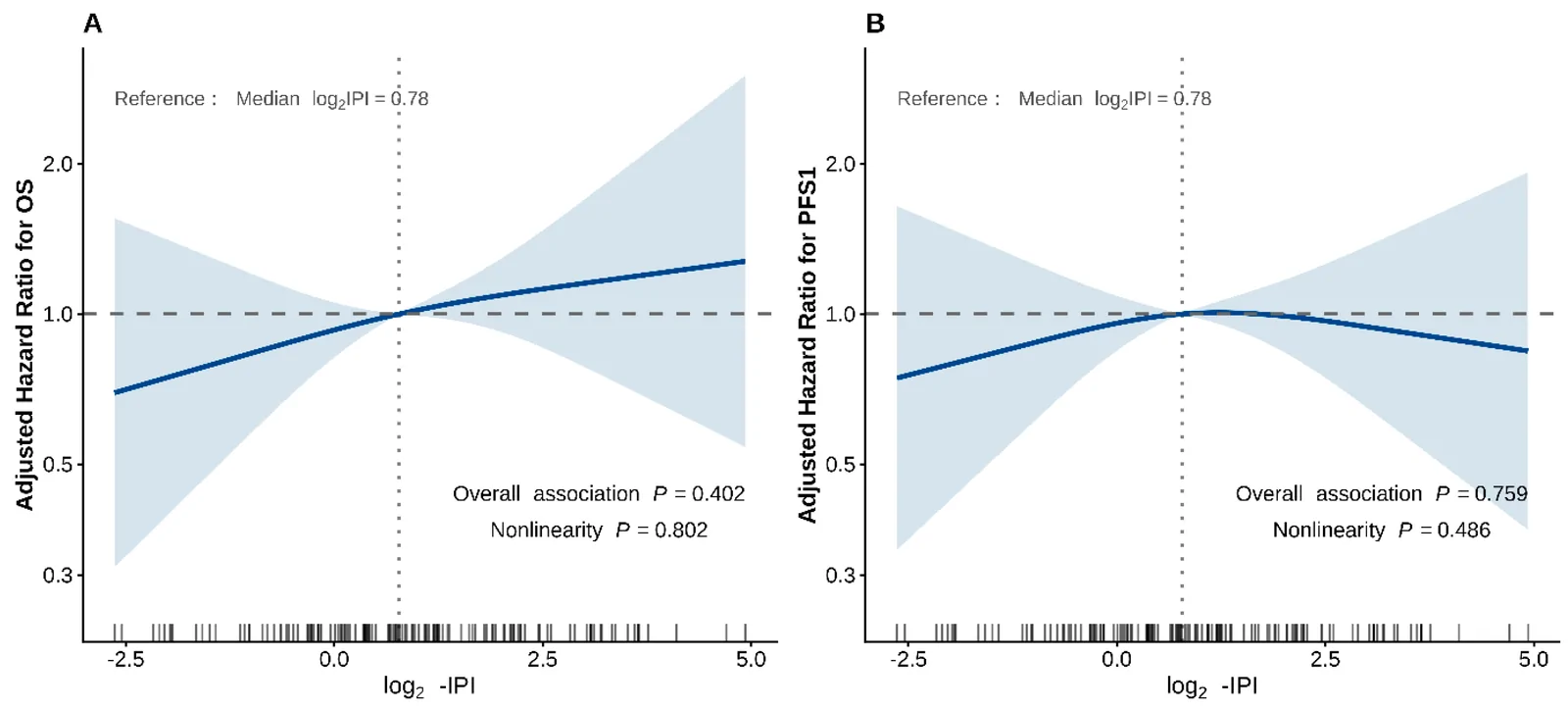
Adjusted continuous associations of baseline inflammatory prognostic index with overall and first-line progression-free survival. (**A**) shows the association between log_2_-transformed IPI and OS, and (**B**) shows the association with PFS1. Solid lines represent adjusted HRs, and shaded areas represent 95% CIs. Restricted cubic spline models used three knots at the 10th, 50th, and 90th percentiles, with the median log_2_(IPI) value (0.78) as the reference. Models were adjusted for age; sex; ECOG performance status; body mass index; liver, brain, and bone metastases; platinum backbone; first-line atezolizumab exposure; and log_2_-EASIX. *p* values for the overall association and nonlinearity were 0.402 and 0.802 for OS and 0.759 and 0.486 for PFS1, respectively. Estimates at the extremes of the distribution should be interpreted cautiously because of the wide confidence intervals. Abbreviations: CI, confidence interval; EASIX, Endothelial Activation and Stress Index; ECOG, Eastern Cooperative Oncology Group; HR, hazard ratio; IPI, Inflammatory Prognostic Index; OS, overall survival; PFS1, first-line progression-free survival.

In the adjusted restricted cubic spline analyses, baseline IPI showed no statistically significant overall or nonlinear association with OS (*p* for overall association = 0.402; *p* for nonlinearity = 0.802). Similarly, neither the overall nor the nonlinear association between IPI and PFS1 was statistically significant (*p* = 0.759 and *p* = 0.486, respectively) (Figure 2).

## 4. Discussion

Although the clinical course of extensive-stage SCLC varies substantially among patients, prognostic assessment in routine practice largely relies on performance status, metastatic disease characteristics, and treatment-related clinical factors [20,21]. Nevertheless, there remains a need for readily available and objective biomarkers that can provide additional prognostic information beyond these clinical features. In this study, we compared EASIX, which reflects endothelial activation and cellular stress, with IPI, which integrates systemic inflammation and nutritional status, within the same patient cohort to evaluate the prognostic relevance of these two distinct biological axes. Our findings indicate that EASIX provides prognostic information independent of clinical variables, particularly for OS, whereas IPI does not offer a comparable contribution. The weak correlation between EASIX and IPI, together with their differing associations with survival outcomes, suggests that these indices may capture distinct biological dimensions and that endothelial stress-related processes may have a separate prognostic role in the clinical course of extensive-stage SCLC.

EASIX was initially developed to evaluate complications related to endothelial activation and stress in hematological diseases, and subsequent studies have suggested that it may also have prognostic relevance in hematological malignancies and various solid tumors [12,13,14,16]. In a previous study of patients with extensive-stage SCLC, higher EASIX values were associated with shorter OS [15]. EASIX was modeled as a continuous measure without applying a predefined threshold. A twofold increase in the score remained independently associated with a greater risk of death, consistent with previous evidence. However, the absence of an independent association between EASIX and PFS1 in the overall cohort suggests that its prognostic relevance may not be consistent across different survival outcomes. Moreover, the simultaneous evaluation of EASIX and IPI within the same model and the additional prognostic information provided by EASIX when added to the clinical model for OS distinguish the present study from previous investigations. However, the absolute increase in the C-index from 0.619 to 0.650 was modest. Therefore, although this finding supports incremental prognostic information from EASIX, it should not by itself be interpreted as demonstrating a clinically meaningful improvement or established utility for clinical decision-making.

IPI was originally proposed in non-small-cell lung cancer to combine measures of systemic inflammation and nutritional status, with higher scores being linked to poorer survival [18]. A subsequent study in extensive-stage SCLC reported an association between elevated IPI values and shorter OS that persisted after multivariable adjustment [19]. By contrast, IPI was not independently associated with OS or PFS1 in our cohort and did not improve the prognostic contribution of the clinical model. This discrepancy may reflect differences in cohort composition, treatment patterns, the analytical handling of IPI, and covariate selection. Evaluating IPI together with EASIX also allowed the inflammation–nutrition axis to be examined after accounting for the information captured by the endothelial stress index.

In our study, established clinical prognostic factors also retained their associations with survival outcomes. An ECOG performance status of 2–3 emerged as an adverse factor for both OS and PFS1, whereas liver metastasis was independently associated with a higher risk of death but not with PFS1. These findings are consistent with previous real-world studies reporting poorer survival outcomes among patients with impaired performance status and liver involvement [20,21]. Poor performance status may reflect greater disease burden, increased symptom severity, reduced physiological reserve, and limited treatment tolerance, whereas liver metastasis may indicate a more aggressive disease phenotype and greater systemic tumor burden. The association between EASIX and OS remained after adjustment for established clinical variables, suggesting that the index may capture prognostic information beyond performance status and metastatic disease characteristics.

First-line atezolizumab use was independently associated with lower risks of both death and progression or death in our study. This finding is broadly consistent with previous randomized and real-world studies demonstrating improved survival outcomes with the addition of atezolizumab to platinum–etoposide [22,23,24]. Although the pivotal IMpower133 trial evaluated atezolizumab in combination with carboplatin–etoposide, a proportion of patients in our real-world cohort received atezolizumab with cisplatin–etoposide according to routine clinical practice. Platinum backbone was therefore incorporated into the multivariable models, and platinum-restricted and interaction analyses were performed. Notably, the adverse associations of EASIX with OS and PFS1 were observed among patients receiving atezolizumab, and the interactions between EASIX and atezolizumab exposure were statistically significant for both survival outcomes. These findings suggest that the prognostic information captured by EASIX may vary according to treatment context. However, these exploratory results are insufficient to establish EASIX as a predictive biomarker of benefit from atezolizumab and require validation in independent cohorts.

Additional analyses indicated that the prognostic relevance of EASIX was not equally pronounced under all analytical conditions. The persistence of the association across most sensitivity analyses indicates that the main finding was not excessively dependent on specific modeling choices. However, its attenuation after accounting for treatment-start year suggests that temporal changes in treatment practice and follow-up maturity, as well as residual confounding, cannot be completely excluded. Calendar year may have acted as a proxy for unmeasured time-related differences in treatment selection and patient management during the study period, as well as for the shorter potential follow-up of patients treated in later years. Accordingly, the attenuation of the EASIX–OS association after additional adjustment for treatment-start year suggests that the primary association may have been partly influenced by unmeasured temporal factors and supports a cautious interpretation of the main EASIX finding. In addition, the nonlinear pattern observed in the spline analyses indicates that the association between EASIX and survival may not be fully captured by a single linear effect estimate. Significant interactions were also observed between EASIX and platinum backbone, with the associations of EASIX with OS and PFS1 being evident in the carboplatin–etoposide group but not in the cisplatin–etoposide group. However, because platinum selection in routine clinical practice is guided by renal function, performance status, comorbidities, and other patient factors, these subgroup findings should not be interpreted as evidence that EASIX predicts differential benefit from a particular platinum regimen. Overall, the results suggest that EASIX may provide additional prognostic information, but confirmation in larger independent cohorts is required.

This study has several limitations. Owing to its retrospective, single-center design, the potential influence of unmeasured clinical factors on the results could not be completely excluded. Because atezolizumab use and platinum selection were determined according to routine clinical practice, the subgroup and interaction findings related to these treatments may have been influenced by patients’ clinical characteristics. In addition, the limited sample size reduced the precision of the analyses, particularly within the treatment subgroups; therefore, these findings should be considered exploratory. EASIX and IPI were calculated using a single pretreatment laboratory assessment, and changes in these indices during treatment could not be evaluated. Finally, the findings were not validated in an independent cohort. Therefore, confirmation in larger, multicenter studies is required.

## 5. Conclusions

In this direct comparison of EASIX and IPI within the same cohort, baseline EASIX was independently associated with OS and provided additional prognostic information beyond established clinical factors. However, the EASIX–OS association was attenuated and was no longer statistically significant after additional adjustment for treatment-start year, and the primary finding should therefore be interpreted cautiously. No independent association was observed between IPI and either OS or PFS1. EASIX may therefore provide additional information for routine prognostic assessment in extensive-stage SCLC. Nevertheless, both its prognostic value and the exploratory treatment-context findings require confirmation in larger, independent multicenter cohorts.

## Figures and Tables

**Table 1:** Baseline demographic, clinical, disease, treatment, and laboratory characteristics of the study population.

Characteristic	Overall (N = 129)	Chemotherapy Alone (n = 77)	Chemotherapy Plus Atezolizumab (n = 52)	Absolute SMD
**Demographic and clinical characteristics**				
Age, years	64 (59–69)	64 (58–69)	62.5 (59–69)	0.080
Age group, n (%)				0.002
<65 years	72 (55.8)	43 (55.8)	29 (55.8)	
≥65 years	57 (44.2)	34 (44.2)	23 (44.2)	
Sex, n (%)				0.478
Female	23 (17.8)	8 (10.4)	15 (28.8)	
Male	106 (82.2)	69 (89.6)	37 (71.2)	
Body mass index, kg/m^2^	26.0 (23.7–29.1)	25.3 (23.6–28.2)	26.8 (24.0–30.4)	0.222
Smoking status, n (%)				0.024
Never smoked	18 (14.0)	11 (14.3)	7 (13.5)	
Current or former smoker	111 (86.0)	66 (85.7)	45 (86.5)	
Smoking exposure, n (%)				0.081
<30 pack-years or never smoked	35 (27.1)	22 (28.6)	13 (25.0)	
≥30 pack-years	94 (72.9)	55 (71.4)	39 (75.0)	
ECOG performance status, n (%)				0.145
0–1	99 (76.7)	61 (79.2)	38 (73.1)	
2–3	30 (23.3)	16 (20.8)	14 (26.9)	
Metastatic disease characteristics				
Brain metastasis, n (%)				0.188
Absent	98 (76.0)	61 (79.2)	37 (71.2)	
Present	31 (24.0)	16 (20.8)	15 (28.8)	
Liver metastasis, n (%)				0.050
Absent	85 (65.9)	50 (64.9)	35 (67.3)	
Present	44 (34.1)	27 (35.1)	17 (32.7)	
Bone metastasis, n (%)				0.077
Absent	65 (50.4)	40 (51.9)	25 (48.1)	
Present	64 (49.6)	37 (48.1)	27 (51.9)	
Pleural metastasis, n (%)				0.069
Absent	111 (86.0)	67 (87.0)	44 (84.6)	
Present	18 (14.0)	10 (13.0)	8 (15.4)	
Adrenal metastasis, n (%)				0.006
Absent	104 (80.6)	62 (80.5)	42 (80.8)	
Present	25 (19.4)	15 (19.5)	10 (19.2)	
Lymph node metastasis, n (%)				0.146
Absent	45 (34.9)	29 (37.7)	16 (30.8)	
Present	84 (65.1)	48 (62.3)	36 (69.2)	
First-line treatment characteristics				
Platinum backbone, n (%)				0.625
Cisplatin–etoposide	60 (46.5)	45 (58.4)	15 (28.8)	
Carboplatin–etoposide	69 (53.5)	32 (41.6)	37 (71.2)	
Baseline laboratory parameters				
Hemoglobin, g/dL	13.0 (12.0–14.3)	13.0 (12.1–14.6)	13.0 (12.0–13.8)	0.136
Neutrophil count, ×10^9^/L	6.4 (4.9–8.0)	6.1 (4.9–7.4)	6.5 (4.9–9.2)	0.361
Lymphocyte count, ×10^9^/L	1.71 (1.11–2.03)	1.70 (1.13–2.02)	1.73 (1.09–2.03)	0.048
Platelet count, ×10^9^/L	294 (226–361)	301 (230–361)	283 (216–364)	0.131
Lactate dehydrogenase, U/L	298 (227–471)	298 (232–471)	294 (204–457)	0.264
Creatinine, mg/dL	0.80 (0.70–0.92)	0.80 (0.70–0.90)	0.80 (0.70–1.00)	0.057
C-reactive protein, mg/L	20.0 (10.0–60.0)	31.0 (12.0–79.0)	14.0 (7.8–27.3)	0.600
Albumin, g/L	38.0 (33.0–41.0)	37.0 (32.0–41.0)	38.5 (35.8–41.3)	0.198
Neutrophil-to-lymphocyte ratio	4.10 (2.66–6.69)	3.50 (2.60–6.22)	4.36 (2.83–6.97)	0.101
Endothelial Activation and Stress Index	0.85 (0.55–1.49)	0.87 (0.54–1.50)	0.82 (0.56–1.42)	0.248
Inflammatory Prognostic Index	2.18 (0.83–7.71)	2.96 (1.14–9.12)	1.49 (0.73–4.12)	0.150

Abbreviations: ECOG, Eastern Cooperative Oncology Group; SMD, standardized mean difference. Footnotes: Data are presented as median (interquartile range) unless otherwise indicated. Categorical variables are presented as number (percentage). Baseline laboratory measurements were defined as the closest available values obtained within 7 days before initiation of first-line systemic therapy. Absolute standardized mean differences (SMDs) were used to quantify baseline differences between the treatment groups; values < 0.10 were considered negligible. SMDs are shown only on the parent variable rows. No hypothesis-testing *p* values were calculated for baseline group comparisons. EASIX and IPI are presented in their original scales.

**Table 2:** Univariable and multivariable Cox proportional hazards regression analyses for overall survival.

Variable	Univariable HR (95% CI)	*p* Value	Multivariable HR (95% CI)	*p* Value
Age, per 10-year increase	1.01 (0.78–1.32)	0.942	0.92 (0.69–1.21)	0.543
Male sex, vs. female	1.32 (0.78–2.22)	0.299	1.11 (0.62–2.00)	0.719
ECOG performance status 2–3, vs. 0–1	1.73 (1.14–2.64)	0.010	1.98 (1.27–3.10)	0.003
Body mass index, per 5-kg/m^2^ increase	0.95 (0.76–1.18)	0.623	0.99 (0.79–1.24)	0.929
Liver metastasis, present vs. absent	1.78 (1.20–2.63)	0.004	1.68 (1.05–2.70)	0.031
Brain metastasis, present vs. absent	0.97 (0.63–1.49)	0.888	1.12 (0.71–1.77)	0.615
Bone metastasis, present vs. absent	1.03 (0.71–1.49)	0.869	1.03 (0.69–1.53)	0.890
Carboplatin–etoposide, vs. cisplatin–etoposide	1.04 (0.72–1.50)	0.824	1.36 (0.89–2.08)	0.152
First-line atezolizumab, yes vs. no	0.65 (0.44–0.96)	0.029	0.62 (0.40–0.97)	0.036
log_2_-EASIX, per doubling	1.18 (1.06–1.31)	0.003	1.14 (1.01–1.28)	0.019
log_2_-IPI, per doubling	1.08 (1.00–1.16)	0.057	1.06 (0.97–1.15)	0.179

Abbreviations: CI, confidence interval; EASIX, Endothelial Activation and Stress Index; ECOG, Eastern Cooperative Oncology Group; HR, hazard ratio; IPI, Inflammatory Prognostic Index. Footnotes: Analyses included 129 patients and 116 deaths; no missing values were present for the variables included in the primary model. The multivariable model simultaneously included all variables shown in the table. Age and body mass index were modeled continuously, with HRs corresponding to 10-year and 5-kg/m^2^ increases, respectively. Reference categories were female sex, ECOG performance status 0–1, absence of the specified metastatic site, cisplatin–etoposide, and no first-line atezolizumab. EASIX and IPI were entered simultaneously and log_2_-transformed before modeling; therefore, their HRs represent the relative change in the hazard of death associated with each doubling of the respective index value.

**Table 3:** Univariable and multivariable Cox proportional hazards regression analyses for first-line progression-free survival.

Variable	Univariable HR (95% CI)	*p* Value	Multivariable HR (95% CI)	*p* Value
Age, per 10-year increase	0.93 (0.72–1.21)	0.596	0.88 (0.66–1.15)	0.347
Male sex, vs. female	1.35 (0.82–2.23)	0.243	1.07 (0.60–1.91)	0.820
ECOG performance status 2–3, vs. 0–1	1.63 (1.08–2.48)	0.021	2.02 (1.28–3.17)	0.002
Body mass index, per 5-kg/m^2^ increase	0.98 (0.80–1.21)	0.877	1.06 (0.85–1.34)	0.602
Liver metastasis, present vs. absent	1.33 (0.91–1.94)	0.141	1.52 (0.94–2.45)	0.084
Brain metastasis, present vs. absent	1.03 (0.68–1.56)	0.893	1.20 (0.77–1.89)	0.425
Bone metastasis, present vs. absent	0.91 (0.63–1.29)	0.588	0.89 (0.60–1.32)	0.576
Carboplatin–etoposide, vs. cisplatin–etoposide	0.98 (0.69–1.40)	0.913	1.53 (0.99–2.35)	0.055
First-line atezolizumab, yes vs. no	0.50 (0.34–0.74)	<0.001	0.41 (0.26–0.64)	<0.001
log_2_-EASIX, per doubling	1.13 (1.03–1.23)	0.008	1.01 (0.88–1.17)	0.850
log_2_-IPI, per doubling	1.04 (0.97–1.12)	0.234	1.01 (0.93–1.09)	0.786

Abbreviations: CI, confidence interval; EASIX, Endothelial Activation and Stress Index; ECOG, Eastern Cooperative Oncology Group; HR, hazard ratio; IPI, Inflammatory Prognostic Index. Footnotes: Analyses included 129 patients and 123 progression-or-death events; no missing values were present for the variables included in the primary model. The multivariable model simultaneously included all variables shown in the table. Age and body mass index were modeled continuously, with HRs corresponding to 10-year and 5-kg/m^2^ increases, respectively. Reference categories were female sex, ECOG performance status 0–1, absence of the specified metastatic site, cisplatin–etoposide, and no first-line atezolizumab. EASIX and IPI were entered simultaneously and log_2_-transformed before modeling; therefore, their HRs represent the relative change in the hazard of progression or death associated with each doubling of the respective index value.

## Data Availability

The datasets generated and/or analyzed during the current study are not publicly available due to institutional and patient privacy regulations but are available from the corresponding author upon reasonable request.

## Supplementary Materials

The following supporting information can be downloaded at: https://www.mdpi.com/article/10.3390/cancers18172888/s1, Table S1: Complete univariable Cox regression analyses for overall and first-line progression-free survival; Table S2: Alternative multivariable Cox proportional hazard regression models for overall survival; Table S3: Alternative multivariable Cox proportional hazard regression models for first-line progression-free survival; Table S4: Sensitivity analyses of the continuous associations of EASIX and IPI with overall and first-line progression-free survival; Table S5: Sensitivity analyses of EASIX and IPI restricted to patients receiving first-line atezolizumab; Table S6: Exploratory interaction analyses according to first-line atezolizumab exposure and platinum backbone; Table S7: Follow-up maturity and time-specific survival estimates in the overall cohort and according to first-line treatment; Figure S1: Distributions of EASIX and IPI values before and after log_2_ transformation; Figure S2: Spearman correlation matrix for EASIX, IPI, and selected baseline clinical and laboratory variables.
