# Supplementary material for: Prognostic and Treatment-Context Associations of EASIX and IPI in Extensive-Stage Small-Cell Lung Cancer Patients Receiving First-Line Therapy
Source: Cancers (Basel). 2026 Sep 6;18(17):2888. doi: 10.3390/cancers18172888 (PMC13565404; doi:10.3390/cancers18172888)
Supplement: Supplementary file 1 [file cancers-18-02888-s001.zip › cancers-4514217-supplementary.pdf]

Prognostic and Treatment-Context Associations of EASIX and IPI in Extensive-Stage Small-Cell Lung Cancer Patients Receiving First-Line Therapy

Hatice Ayyıldız Sevim, MD*, Galip Can Uyar, MD, Güner Akgüner, MD, Hayriye Şahinli, MD

Supplementary Table S1. Complete univariable Cox regression analyses for overall and first-line progression-free survival

Variable	OS HR (95% CI)	OS *P* value	PFS1 HR (95% CI)	PFS1 *P* value
Demographic and clinical characteristics				
Age, per 10-year increase	1.01 (0.78–1.32)	0.942	0.93 (0.72–1.21)	0.596
Age ≥65 years, vs <65 years	1.30 (0.90–1.88)	0.164	1.07 (0.75–1.53)	0.702
Male sex, vs female	1.32 (0.78–2.22)	0.299	1.35 (0.82–2.23)	0.243
ECOG performance status 2–3, vs 0–1	1.73 (1.14–2.64)	0.010	1.63 (1.08–2.48)	0.021
Body mass index, per 5-kg/m² increase	0.95 (0.76–1.18)	0.623	0.98 (0.80–1.21)	0.877
Current or former smoker, vs never smoker	0.75 (0.44–1.29)	0.301	0.90 (0.54–1.51)	0.691
Smoking exposure ≥30 pack-years, vs <30 pack-years or never smoker	0.67 (0.44–1.01)	0.057	0.84 (0.56–1.26)	0.406
Metastatic disease characteristics				
Brain metastasis, present vs absent	0.97 (0.63–1.49)	0.888	1.03 (0.68–1.56)	0.893
Liver metastasis, present vs absent	1.78 (1.20–2.63)	0.004	1.33 (0.91–1.94)	0.141
Bone metastasis, present vs absent	1.03 (0.71–1.49)	0.869	0.91 (0.63–1.29)	0.588
Pleural metastasis, present vs absent	1.08 (0.64–1.80)	0.780	1.07 (0.64–1.79)	0.803
Adrenal metastasis, present vs absent	1.80 (1.13–2.86)	0.013	1.78 (1.12–2.82)	0.014
Lymph node metastasis, present vs absent	1.08 (0.73–1.59)	0.697	0.94 (0.65–1.36)	0.746
Number of metastatic sites, per 1-site increase	1.29 (1.07–1.56)	0.009	1.14 (0.94–1.38)	0.195
First-line treatment characteristics				
Carboplatin–etoposide, vs cisplatin–etoposide	1.04 (0.72–1.50)	0.824	0.98 (0.69–1.40)	0.913
First-line atezolizumab, yes vs no	0.65 (0.44–0.96)	0.029	0.50 (0.34–0.74)	<0.001
Baseline laboratory parameters				
Hemoglobin, per 1-g/dL increase	0.96 (0.87–1.05)	0.335	1.00 (0.92–1.09)	0.979
Neutrophil count, per 1 ×10⁹/L increase	1.01 (0.95–1.08)	0.769	1.01 (0.95–1.07)	0.773
Lymphocyte count, per 1 ×10⁹/L increase	0.90 (0.69–1.17)	0.430	0.94 (0.74–1.20)	0.636
Neutrophil-to-lymphocyte ratio, per 1-unit increase	1.00 (0.97–1.03)	0.906	1.00 (0.97–1.03)	0.990
Platelet count, per 50 ×10⁹/L increase	0.97 (0.89–1.06)	0.532	0.97 (0.90–1.06)	0.533
Lactate dehydrogenase, per 100-U/L increase	1.01 (0.99–1.03)	0.454	1.00 (0.98–1.02)	0.986
Creatinine, per 0.1-mg/dL increase	1.01 (0.92–1.11)	0.766	0.98 (0.90–1.07)	0.621
C-reactive protein, per 10-mg/L increase	1.03 (0.99–1.07)	0.134	1.01 (0.97–1.05)	0.526
Albumin, per 5-g/L increase	0.81 (0.70–0.94)	0.006	0.89 (0.77–1.03)	0.116
Composite prognostic indices				
$\log_2$-EASIX, per doubling	1.18 (1.06–1.31)	0.003	1.13 (1.03–1.23)	0.008
$\log_2$-IPI, per doubling	1.08 (1.00–1.16)	0.057	1.04 (0.97–1.12)	0.234

Abbreviations: BMI, body mass index; CI, confidence interval; CRP, C-reactive protein; EASIX, Endothelial Activation and Stress Index; ECOG, Eastern Cooperative Oncology Group; HR, hazard ratio; IPI, Inflammatory Prognostic Index; LDH, lactate dehydrogenase; NLR, neutrophil-to-lymphocyte ratio; OS, overall survival; PFS1, first-line progression-free survival.

Footnotes: Analyses included all 129 patients, 116 deaths, and 123 PFS1 events. No missing values were present for the variables included in this table. OS was calculated from initiation of first-line systemic therapy to death from any cause; patients alive at the data cutoff were censored at the date of last follow-up. PFS1 was calculated from initiation of first-line systemic therapy to radiographic or clinical disease progression or death from any cause, whichever occurred first; patients without an event were censored at the date of the last adequate disease assessment. HRs and 95% CIs were estimated in separate univariable Cox proportional hazards models, with the Efron method used to handle tied event times. Age and BMI were rescaled so that HRs correspond to a 10-year and 5-kg/m² increase, respectively. Hemoglobin, neutrophil count, lymphocyte count, NLR, platelet count, LDH, creatinine, CRP, and albumin were modeled continuously using the increments specified in the variable labels. The number of

metastatic sites was calculated as the sum of brain, liver, bone, pleural, adrenal, and lymph-node metastatic sites documented at baseline. Reference categories were age <65 years, female sex, ECOG performance status 0–1, never smoking, smoking exposure <30 pack-years or never smoking, absence of the specified metastatic site, cisplatin–etoposide, and no first-line atezolizumab. EASIX was recalculated as LDH (U/L) × serum creatinine (mg/dL) / platelet count (×10^9/L), and IPI as CRP (mg/L) × NLR / serum albumin (g/L). EASIX and IPI were log_2-transformed; therefore, their HRs represent the relative change in the hazard associated with each doubling of the respective index. Palliative radiotherapy was not analyzed because it could have been administered after initiation of systemic therapy and was therefore not considered a baseline prognostic covariate. Wald 95% CIs and two-sided *P* values are reported; *P*<0.05 was considered statistically significant. No multiplicity adjustment was applied because these analyses were descriptive and exploratory.

Supplementary Table S2. Alternative multivariable Cox proportional hazards regression models for overall survival

Panel A. Adjusted associations across alternative models

Variable	Model A HR (95% CI)	*P* value	Model B HR (95% CI)	*P* value	Model C HR (95% CI)	*P* value	Model D HR (95% CI)	*P* value
Age, per 10-year increase	0.89 (0.68–1.17)	0.412	0.92 (0.70–1.22)	0.583	0.89 (0.67–1.16)	0.378	0.92 (0.69–1.21)	0.543
Male sex, vs female	1.15 (0.65–2.06)	0.627	1.18 (0.66–2.10)	0.577	1.09 (0.61–1.95)	0.776	1.11 (0.62–2.00)	0.719
ECOG performance status 2–3, vs 0–1	1.97 (1.25–3.08)	0.003	2.00 (1.27–3.12)	0.003	1.96 (1.25–3.07)	0.003	1.98 (1.27–3.10)	0.003
Body mass index, per 5-kg/m² increase	0.98 (0.78–1.22)	0.845	1.01 (0.80–1.27)	0.936	0.96 (0.77–1.20)	0.733	0.99 (0.79–1.24)	0.929
Liver metastasis, present vs absent	1.93 (1.28–2.92)	0.002	1.65 (1.03–2.63)	0.037	1.95 (1.29–2.95)	0.001	1.68 (1.05–2.70)	0.031
Brain metastasis, present vs absent	1.09 (0.69–1.71)	0.715	1.10 (0.70–1.74)	0.671	1.11 (0.71–1.76)	0.639	1.12 (0.71–1.77)	0.615
Bone metastasis, present vs absent	1.01 (0.68–1.49)	0.962	1.07 (0.72–1.59)	0.755	0.98 (0.66–1.45)	0.908	1.03 (0.69–1.53)	0.890
Carboplatin–etoposide, vs cisplatin–etoposide	1.40 (0.91–2.14)	0.125	1.37 (0.90–2.10)	0.141	1.38 (0.90–2.10)	0.141	1.36 (0.89–2.08)	0.152
First-line atezolizumab, yes vs no	0.57 (0.37–0.88)	0.012	0.61 (0.39–0.94)	0.025	0.60 (0.39–0.93)	0.022	0.62 (0.40–0.97)	0.036
log_2-EASIX, per doubling	—	—	1.19 (1.05–1.35)	0.006	—	—	1.14 (1.01–1.28)	0.019
log_2-IPI, per doubling	—	—	—	—	1.06 (0.98–1.15)	0.121	1.06 (0.97–1.15)	0.179

Panel B. Model fit and discrimination

Model	Covariate specification	No. of parameters	Log-likelihood	AIC	Harrell C-index (95% CI)
Model A	Clinical covariates	9	−453.64	925.28	0.619 (0.555–0.678)
Model B	Clinical covariates + log_2-EASIX	10	−449.89	919.78	0.650 (0.589–0.708)
Model C	Clinical covariates + log_2-IPI	10	−452.45	924.89	0.626 (0.560–0.690)
Model D	Clinical covariates + log_2-EASIX + log_2-IPI	11	−449.30	920.60	0.653 (0.591–0.711)

Panel C. Nested likelihood-ratio tests

Model comparison	Likelihood-ratio χ^2	df	*P* value
Model B vs Model A	7.50	1	**0.006**
Model C vs Model A	2.39	1	0.122
Model D vs Model A	8.68	2	**0.013**
Model D vs Model B	1.18	1	0.277
Model D vs Model C	6.30	1	**0.012**

Abbreviations: AIC, Akaike information criterion; BMI, body mass index; CI, confidence interval; EASIX, Endothelial Activation and Stress Index; ECOG, Eastern Cooperative Oncology Group; HR, hazard ratio; IPI, Inflammatory Prognostic Index; OS, overall survival.

Footnotes: All models included 129 patients and 116 deaths, with complete data for all modeled covariates. Model A included age, sex, ECOG performance status, BMI, liver metastasis, brain metastasis, bone metastasis, platinum backbone, and first-line atezolizumab exposure. Model B added log_2-EASIX to Model A; Model C added log_2-IPI to Model A; and Model D added both log_2-EASIX and log_2-IPI to Model A. Covariates were selected a priori according to clinical relevance and the study objectives, without stepwise or univariable significance-based selection. Age and BMI were modeled continuously and rescaled so that HRs correspond to a 10-year and 5-kg/m² increase, respectively. Reference categories were female sex, ECOG performance status 0–1, absence of the specified metastatic site, cisplatin–etoposide, and no first-line atezolizumab. EASIX and IPI were recalculated from their source laboratory variables and log_2-transformed; their HRs therefore represent the relative change in the hazard of death associated with each doubling of the respective index. Cox models used the Efron method for tied event times. AIC was calculated as -2 log-likelihood + $2k$, where k denotes the number of regression parameters; lower AIC values indicate better relative model fit. Harrell C-indices were calculated from the fitted model linear predictors. Percentile 95% CIs were obtained from 1000 patient-level nonparametric bootstrap resamples of the observed survival outcomes and corresponding model linear predictors, using a fixed random seed. Likelihood-ratio tests compared

nested models. Wald 95% CIs and two-sided *P* values are reported. These analyses evaluate comparative and incremental prognostic value and were not intended to develop or validate a clinical prediction model.

Supplementary Table S3. Alternative multivariable Cox proportional hazards regression models for first-line progression-free survival

Panel A. Adjusted associations across alternative models

Variable	Model A HR (95% CI)	*P* value	Model B HR (95% CI)	*P* value	Model C HR (95% CI)	*P* value	Model D HR (95% CI)	*P* value
Age, per 10-year increase	0.87 (0.67–1.14)	0.313	0.88 (0.67–1.16)	0.352	0.87 (0.67–1.14)	0.312	0.88 (0.66–1.15)	0.347
Male sex, vs female	1.09 (0.61–1.93)	0.775	1.08 (0.61–1.93)	0.783	1.07 (0.60–1.92)	0.817	1.07 (0.60–1.91)	0.820
ECOG performance status 2–3, vs 0–1	2.02 (1.28–3.18)	0.002	2.02 (1.28–3.18)	0.002	2.02 (1.28–3.17)	0.002	2.02 (1.28–3.17)	0.002
Body mass index, per 5-kg/m² increase	1.06 (0.85–1.33)	0.595	1.07 (0.85–1.34)	0.576	1.06 (0.85–1.33)	0.619	1.06 (0.85–1.34)	0.602
Liver metastasis, present vs absent	1.55 (1.03–2.34)	0.036	1.51 (0.94–2.42)	0.087	1.56 (1.03–2.35)	0.035	1.52 (0.94–2.45)	0.084
Brain metastasis, present vs absent	1.19 (0.76–1.87)	0.439	1.19 (0.76–1.87)	0.438	1.20 (0.77–1.89)	0.424	1.20 (0.77–1.89)	0.425
Bone metastasis, present vs absent	0.88 (0.60–1.30)	0.530	0.90 (0.60–1.33)	0.582	0.89 (0.60–1.30)	0.531	0.89 (0.60–1.32)	0.575
Carboplatin–etoposide, vs cisplatin–etoposide	1.53 (1.00–2.36)	0.052	1.53 (0.99–2.35)	0.056	1.53 (1.00–2.36)	0.052	1.53 (0.99–2.35)	0.055
First-line atezolizumab, yes vs no	0.40 (0.25–0.62)	<0.001	0.40 (0.25–0.63)	<0.001	0.40 (0.26–0.63)	<0.001	0.41 (0.26–0.64)	<0.001
log_2-EASIX, per doubling	—	—	1.02 (0.89–1.17)	0.817	—	—	1.01 (0.88–1.17)	0.850
log_2-IPI, per doubling	—	—	—	—	1.01 (0.94–1.09)	0.763	1.01 (0.93–1.09)	0.786

Panel B. Model fit and discrimination

Model	Covariate specification	No. of parameters	Log-likelihood	AIC	Harrell C-index (95% CI)
Model A	Clinical covariates	9	−468.44	954.89	0.653 (0.591–0.705)
Model B	Clinical covariates + log_2-EASIX	10	−468.42	956.83	0.652 (0.592–0.704)
Model C	Clinical covariates + log_2-IPI	10	−468.40	956.80	0.653 (0.592–0.705)
Model D	Clinical covariates + log_2-EASIX + log_2-IPI	11	−468.38	958.76	0.652 (0.590–0.704)

Panel C. Nested likelihood-ratio tests

Model comparison	Likelihood-ratio χ^2	df	*P* value
Model B vs Model A	0.05	1	0.818
Model C vs Model A	0.09	1	0.763
Model D vs Model A	0.13	2	0.939
Model D vs Model B	0.07	1	0.786
Model D vs Model C	0.04	1	0.850

Abbreviations: AIC, Akaike information criterion; BMI, body mass index; CI, confidence interval; EASIX, Endothelial Activation and Stress Index; ECOG, Eastern Cooperative Oncology Group; HR, hazard ratio; IPI, Inflammatory Prognostic Index; PFS1, first-line progression-free survival.

Footnotes: All models included 129 patients and 123 PFS1 events, with complete data for all modeled covariates. PFS1 was calculated from initiation of first-line systemic therapy to radiographic or clinical disease progression or death from any cause, whichever occurred first; patients without an event were censored at the date of the last adequate disease assessment. Model A included age, sex, ECOG performance status, BMI, liver metastasis, brain metastasis, bone metastasis, platinum backbone, and first-line atezolizumab exposure. Model B added log_2-EASIX to Model A; Model C added log_2-IPI to Model A; and Model D added both log_2-EASIX and log_2-IPI to Model A. Covariates were selected a priori according to clinical relevance and the study objectives, without stepwise or univariable significance-based selection. Age and BMI were modeled continuously and rescaled so that HRs correspond to a 10-year and 5-kg/m² increase, respectively. Reference categories were female sex, ECOG performance status 0–1, absence of the specified metastatic site, cisplatin–etoposide, and no first-line atezolizumab. EASIX and IPI were recalculated from their source laboratory variables and log_2-transformed; their HRs therefore represent the relative change in the hazard of progression or death associated with each doubling of the respective index. Cox models used the Efron method for tied event times. AIC was calculated as -2 log-likelihood + $2k$, where k denotes the number of regression parameters; lower AIC values indicate better relative model fit. Harrell C-indices were calculated from the fitted model linear predictors. Percentile 95% CIs were obtained from 1000 patient-level nonparametric bootstrap resamples. Likelihood-ratio tests compared the prespecified nested models. These analyses evaluate comparative and incremental prognostic value and were not intended to develop or validate a clinical prediction model.

Supplementary Table S4. Sensitivity analyses of the continuous associations of EASIX and IPI with overall and first-line progression-free survival

Panel A. Scale and distributional sensitivity analyses

Sensitivity analysis	Marker	Endpoint	N	Events	Adjusted HR (95% CI)	*P* value	Model specification/additional result
Primary model, per doubling	EASIX	OS	129	116	1.14 (1.01–1.28)	0.019	Full prespecified clinical model with both scores
	IPI	OS	129	116	1.06 (0.97–1.15)	0.179	Full prespecified clinical model with both scores
	EASIX	PFS1	129	123	1.01 (0.88–1.17)	0.850	Full prespecified clinical model with both scores
	IPI	PFS1	129	123	1.01 (0.93–1.09)	0.786	Full prespecified clinical model with both scores
Per IQR increase	EASIX	OS	129	116	1.21 (1.01–1.43)	0.019	IQR of $\log_2$-EASIX=1.445
	IPI	OS	129	116	1.20 (0.92–1.55)	0.179	IQR of $\log_2$-IPI=3.224
	EASIX	PFS1	129	123	1.02 (0.83–1.25)	0.850	IQR of $\log_2$-EASIX=1.445
	IPI	PFS1	129	123	1.04 (0.80–1.34)	0.786	IQR of $\log_2$-IPI=3.224
Per 1-SD increase	EASIX	OS	129	116	1.18 (1.01–1.37)	0.019	SD of $\log_2$-EASIX=1.285
	IPI	OS	129	116	1.14 (0.94–1.38)	0.179	SD of $\log_2$-IPI=2.329
	EASIX	PFS1	129	123	1.02 (0.85–1.22)	0.850	SD of $\log_2$-EASIX=1.285
	IPI	PFS1	129	123	1.03 (0.85–1.23)	0.786	SD of $\log_2$-IPI=2.329
Winsorized at 1st/99th percentiles	EASIX	OS	129	116	1.17 (1.01–1.37)	0.048	Raw-score limits=0.319–13.880
	IPI	OS	129	116	1.06 (0.97–1.15)	0.201	Raw-score limits=0.089–96.545
	EASIX	PFS1	129	123	1.09 (0.93–1.28)	0.296	Raw-score limits=0.319–13.880
	IPI	PFS1	129	123	1.01 (0.93–1.09)	0.885	Raw-score limits=0.089–96.545

Panel B. Influence, variance, calendar-time, and metastatic-burden analyses

Sensitivity analysis	Marker	Endpoint	N	Events	Adjusted HR (95% CI)	*P* value	Model specification/additional result
Influence-restricted analysis	EASIX	OS	123	111	1.27 (1.06–1.51)	0.010	Excluded n=6; |DFBETAS|>0.176
	IPI	OS	118	108	1.08 (0.98–1.20)	0.130	Excluded n=11; |DFBETAS|>0.176
	EASIX	PFS1	123	117	1.26 (1.05–1.51)	0.012	Excluded n=6; |DFBETAS|>0.176
	IPI	PFS1	117	113	1.02 (0.92–1.14)	0.681	Excluded n=12; |DFBETAS|>0.176
Additional adjustment for treatment-start year	EASIX	OS	129	116	1.11 (0.96–1.27)	0.151	Treatment year aHR=1.99 (1.44–2.74), *P*<0.001
	IPI	OS	129	116	1.07 (0.99–1.16)	0.093	Treatment year aHR=1.99 (1.44–2.74), *P*<0.001
	EASIX	PFS1	129	123	1.03 (0.89–1.18)	0.729	Treatment year aHR=1.68 (1.23–2.30), *P*=0.001
	IPI	PFS1	129	123	1.01 (0.93–1.09)	0.798	Treatment year aHR=1.68 (1.23–2.30), *P*=0.001
Huber–White robust standard errors	EASIX	OS	129	116	1.14 (1.01–1.28)	0.040	Individual-level sandwich variance
	IPI	OS	129	116	1.06 (0.97–1.15)	0.191	Individual-level sandwich variance
	EASIX	PFS1	129	123	1.01 (0.83–1.23)	0.893	Individual-level sandwich variance
	IPI	PFS1	129	123	1.01 (0.93–1.09)	0.786	Individual-level sandwich variance
Alternative metastatic-burden model	EASIX	OS	129	116	1.14 (1.01–1.29)	0.037	Site count aHR=1.23 (1.00–1.51), *P*=0.050
	IPI	OS	129	116	1.05 (0.97–1.14)	0.245	Site count aHR=1.23 (1.00–1.51), *P*=0.050
	EASIX	PFS1	129	123	1.06 (0.94–1.20)	0.329	Site count aHR=1.11 (0.90–1.37), *P*=0.316
	IPI	PFS1	129	123	1.00 (0.93–1.08)	0.952	Site count aHR=1.11 (0.90–1.37), *P*=0.316

Panel C. Platinum-backbone restricted and time-varying effect analyses

Sensitivity analysis	Marker	Endpoint	N	Events	Adjusted HR (95% CI)	*P* value	Model specification/additional result
Cisplatin–etoposide restricted	EASIX	OS	60	57	0.94 (0.78–1.12)	0.478	Adjusted for age, ECOG, liver metastasis, and atezolizumab
	IPI	OS	60	57	1.05 (0.94–1.18)	0.351	Adjusted for age, ECOG, liver metastasis, and atezolizumab
	EASIX	PFS1	60	60	0.84 (0.69–1.02)	0.085	Adjusted for age, ECOG, liver metastasis, and atezolizumab
	IPI	PFS1	60	60	0.95 (0.85–1.07)	0.396	Adjusted for age, ECOG, liver metastasis, and atezolizumab
Carboplatin–etoposide restricted	EASIX	OS	69	59	1.49 (1.18–1.89)	<0.001	Adjusted for age, ECOG, liver metastasis, and atezolizumab
	IPI	OS	69	59	1.07 (0.95–1.20)	0.255	Adjusted for age, ECOG, liver metastasis, and atezolizumab
	EASIX	PFS1	69	63	1.32 (1.06–1.65)	0.012	Adjusted for age, ECOG, liver metastasis, and atezolizumab
	IPI	PFS1	69	63	1.04 (0.93–1.16)	0.516	Adjusted for age, ECOG, liver metastasis, and atezolizumab
Time-varying EASIX coefficient	EASIX	PFS1	129	123	1.04 (0.90–1.20)	0.606	HR at 6 months; EASIX×log(time/6) ratio=0.84 (0.70–1.01), *P*=0.070

Abbreviations: BMI, body mass index; CI, confidence interval; DFBETAS, standardized difference in beta; EASIX, Endothelial Activation and Stress Index; ECOG, Eastern Cooperative Oncology Group; HR, hazard ratio; IPI, Inflammatory Prognostic Index; IQR, interquartile range; OS, overall survival; PFS1, first-line progression-free survival; SD, standard deviation.

Unless otherwise specified, sensitivity analyses were performed in the full cohort using the prespecified multivariable Cox model adjusted for age, sex, Eastern Cooperative Oncology Group performance status, body mass index, liver metastasis, brain metastasis, bone metastasis, platinum backbone, first-line atezolizumab exposure, $\log_2$-transformed EASIX, and $\log_2$-transformed IPI. EASIX and IPI were retained simultaneously in the primary, rescaled, winsorized, treatment-year-adjusted, robust-variance, and alternative metastatic-burden models. Hazard ratios in the primary analyses represent the relative change in the hazard of the relevant outcome associated with each doubling of the respective index. For the

interquartile-range- and standard-deviation-scaled analyses, the log_2-transformed indices were rescaled according to their observed interquartile ranges and standard deviations, respectively; these analyses represent alternative effect-size presentations and do not alter model fit or statistical significance. For the winsorized analyses, the original EASIX and IPI distributions were truncated at the 1st and 99th percentiles before log_2 transformation. Influence-restricted analyses excluded only patients whose absolute DFBETAS exceeded the prespecified screening threshold of $2/\sqrt{n}=0.176$ for the relevant biomarker and endpoint; these exclusions were used solely as sensitivity analyses and were not applied to the primary cohort. Treatment-start year was entered as a continuous covariate. In the alternative metastatic-burden model, the individual indicators for liver, brain, and bone metastases were replaced by the total number of baseline metastatic sites across the brain, liver, bone, pleura, adrenal glands, and lymph nodes. Robust results were estimated using individual-level Huber–White sandwich standard errors. Because of the smaller sample sizes within the platinum-backbone subgroups, cisplatin–etoposide- and carboplatin–etoposide-restricted analyses used parsimonious, biomarker-specific models adjusted for age, ECOG performance status, liver metastasis, and first-line atezolizumab exposure. The time-varying PFS1 model included a log_2-EASIX × log (time/6 months) interaction term in addition to the complete primary model; consequently, the reported EASIX hazard ratio represents its association at 6 months, whereas the interaction ratio quantifies the change in the EASIX log-hazard ratio over logarithmic follow-up time. Cox proportional hazards models used the Efron method for tied event times. Wald 95% confidence intervals and two-sided P values are reported. No biomarker cut-off, categorization, receiver-operating-characteristic, Youden-index, or Kaplan–Meier analysis based on biomarker groups was performed.

Supplementary Table S5. Sensitivity analyses of EASIX and IPI restricted to patients receiving first-line atezolizumab

Marker	Endpoint	N	Events	Univariable HR (95% CI)	*P* value	Adjusted HR (95% CI)	*P* value
log_2-EASIX, per doubling	OS	52	41	1.67 (1.22–2.28)	0.001	1.63 (1.15–2.30)	0.006
log_2-EASIX, per doubling	PFS1	52	46	1.49 (1.10–2.02)	0.010	1.54 (1.12–2.12)	0.008
log_2-IPI, per doubling	OS	52	41	1.10 (0.97–1.24)	0.150	1.05 (0.91–1.22)	0.490
log_2-IPI, per doubling	PFS1	52	46	1.08 (0.96–1.22)	0.176	1.03 (0.89–1.18)	0.710

Abbreviations: CI, confidence interval; EASIX, Endothelial Activation and Stress Index; ECOG, Eastern Cooperative Oncology Group; HR, hazard ratio; IPI, Inflammatory Prognostic Index; OS, overall survival; PFS1, first-line progression-free survival.

Footnotes: Analyses were restricted to the 52 patients who received first-line atezolizumab. There were 41 deaths for the OS analyses and 46 progression-or-death events for the PFS1 analyses. Complete data were available for all variables included in these models. EASIX and IPI were recalculated from their source laboratory components and log_2-transformed; hazard ratios therefore represent the relative change in the hazard of the relevant outcome associated with each doubling of the respective index. EASIX and IPI were evaluated in separate models and were not entered simultaneously. Each multivariable Cox model was adjusted for age, entered continuously per 10-year increase; ECOG performance status 2–3 vs 0–1; liver metastasis, present vs absent; brain metastasis, present vs absent; and carboplatin–etoposide vs cisplatin–etoposide. The relevant log_2-transformed score was then added as the sixth model parameter. This parsimonious adjustment set was prespecified to limit overfitting in the subgroup, which contained 41 OS events and 46 PFS1 events. Reference categories were ECOG performance status 0–1, absence of the specified metastatic site, and cisplatin–etoposide. OS was measured from initiation of first-line systemic therapy to death from any cause. PFS1 was measured from initiation of first-line systemic therapy to radiographic or clinical progression or death from any cause, whichever occurred first. Cox proportional hazards models used the Efron method for tied event times. Wald 95% confidence intervals and two-sided *P* values are reported. Because this was a treatment-restricted sensitivity analysis with a limited sample size, the estimates should be interpreted as exploratory. No biomarker cut-off, categorization, receiver-operating-characteristic, Youden-index, or Kaplan–Meier analysis based on biomarker groups was performed.

Supplementary Table S6. Exploratory interaction analyses according to first-line atezolizumab exposure and platinum backbone

**Panel A. Interaction according to first-line atezolizumab exposure**							
**Endpoint**	**Marker**	**No atezolizumab, N/events**	**Atezolizumab, N/events**	**Marker HR in no-atezolizumab group (95% CI)**	**Marker HR in atezolizumab group (95% CI)**	**Interaction HR (95% CI)**	***P* for interaction**
OS	log_2-EASIX, per doubling	77/75	52/41	1.05 (0.91–1.22)	1.55 (1.14–2.12)	1.48 (1.07–2.05)	0.019
PFS1	log_2-EASIX, per doubling	77/77	52/46	0.96 (0.82–1.12)	1.44 (1.05–1.97)	1.51 (1.08–2.10)	0.016
OS	log_2-IPI, per doubling	77/75	52/41	1.06 (0.95–1.17)	1.08 (0.95–1.23)	1.02 (0.86–1.21)	0.795
PFS1	log_2-IPI, per doubling	77/77	52/46	0.97 (0.87–1.07)	1.08 (0.96–1.22)	1.12 (0.95–1.32)	0.177
**Panel B. Interaction according to platinum backbone**							

Endpoint	Marker	Cisplatin–etoposide, N/events	Carboplatin–etoposide, N/events	Marker HR in cisplatin group (95% CI)	Marker HR in carboplatin group (95% CI)	Interaction HR (95% CI)	*P* for interaction
OS	$\log_2$-EASIX, per doubling	60/57	69/59	0.98 (0.83–1.17)	1.44 (1.16–1.79)	1.46 (1.13–1.89)	0.004
PFS1	$\log_2$-EASIX, per doubling	60/60	69/63	0.88 (0.74–1.05)	1.37 (1.10–1.70)	1.56 (1.20–2.03)	<0.001
OS	$\log_2$-IPI, per doubling	60/57	69/59	1.05 (0.94–1.18)	1.08 (0.96–1.21)	1.03 (0.87–1.21)	0.759
PFS1	$\log_2$-IPI, per doubling	60/60	69/63	0.95 (0.85–1.06)	1.08 (0.97–1.20)	1.14 (0.97–1.33)	0.109

Abbreviations: BMI, body mass index; CI, confidence interval; EASIX, Endothelial Activation and Stress Index; ECOG, Eastern Cooperative Oncology Group; HR, hazard ratio; IPI, Inflammatory Prognostic Index; OS, overall survival; PFS1, first-line progression-free survival.

Footnotes: Interaction analyses included all 129 patients, 116 deaths, and 123 PFS1 events. For analyses according to first-line atezolizumab exposure, the no-atezolizumab group comprised 77 patients with 75 deaths and 77 PFS1 events, whereas the atezolizumab group comprised 52 patients with 41 deaths and 46 PFS1 events. For analyses according to platinum backbone, the cisplatin–etoposide group comprised 60 patients with 57 deaths and 60 PFS1 events, whereas the carboplatin–etoposide group comprised 69 patients with 59 deaths and 63 PFS1 events. Complete data were available for all modeled variables. EASIX and IPI were recalculated from their source laboratory components and $\log_2$-transformed; all marker HRs therefore represent the relative change in the hazard of the relevant outcome associated with each doubling of the respective index. EASIX and IPI were evaluated in separate marker-specific Cox models and were not entered simultaneously. Atezolizumab-interaction models included age per 10-year increase, sex, ECOG performance status, BMI per 5-kg/m² increase, liver metastasis, brain metastasis, bone metastasis, platinum backbone, first-line atezolizumab exposure, the relevant marker, and the marker×atezolizumab interaction term. Platinum-interaction models included the same clinical covariates, first-line atezolizumab exposure, platinum backbone, the relevant marker, and the marker×platinum-backbone interaction term. In Panel A, no atezolizumab was the reference exposure; in Panel B, cisplatin–etoposide was the reference platinum backbone. The marker HR in the reference group was estimated from the marker main effect. The marker HR in the comparison group was estimated from the linear combination of the marker and interaction coefficients. The interaction HR represents the ratio of marker HRs between the comparison and reference groups. Cox models used the Efron method for tied event times. Wald 95% confidence intervals and two-sided interaction *P* values are reported. Because eight formal interaction tests were exploratory and no multiplicity adjustment was applied, the results should be interpreted as hypothesis-generating treatment-effect modification analyses and not as evidence of a validated predictive biomarker. No biomarker cut-off, categorization, receiver-operating-characteristic, Youden-index, or Kaplan–Meier analysis based on biomarker groups was performed.

Supplementary Table S7. Follow-up maturity and time-specific survival estimates in the overall cohort and according to first-line treatment

Cohort	N	Reverse KM median follow-up, months (95% CI)	OS events/censored, n	PFS1 events/censored, n	Median OS, months (95% CI)	Median PFS1, months (95% CI)	6-month PFS1, % (95% CI)	12-month PFS1, % (95% CI)	12-month OS, % (95% CI)	18-month OS, % (95% CI)	24-month OS, % (95% CI)
Overall cohort	129	26.91 (19.75–28.25)	116/13	123/6	9.26 (8.28–10.61)	6.64 (6.05–7.10)	61.2 (52.3–69.0)	10.3 (5.7–16.5)	30.2 (22.4–38.3)	13.5 (8.1–20.3)	5.7 (2.3–11.5)
Chemotherapy alone	77	26.91 (17.31–26.91)	75/2	77/0	8.51 (7.46–10.61)	6.18 (5.19–6.90)	54.5 (42.8–64.9)	3.9 (1.0–10.0)	28.6 (19.0–38.9)	7.8 (3.2–15.1)	1.6 (0.1–7.2)
Chemotherapy plus atezolizumab	52	25.59 (16.26–28.25)	41/11	46/6	10.02 (8.44–11.07)	7.72 (6.60–9.43)	71.2 (56.8–81.5)	20.7 (10.5–33.2)	32.6 (19.9–45.9)	23.5 (12.5–36.4)	14.1 (5.3–27.0)

Abbreviations: CI, confidence interval; KM, Kaplan–Meier; NE, not estimable; NR, not reached; OS, overall survival; PFS1, first-line progression-free survival.

Footnotes: The overall cohort included 129 patients, with 116 deaths and 13 OS-censored observations, and 123 progression-or-death events and 6 PFS1-censored observations. The chemotherapy-alone group included 77 patients, with 75 deaths and 2 OS-censored observations, and 77 PFS1 events with no PFS1-censored observations. The chemotherapy-plus-atezolizumab group included 52 patients, with 41 deaths and 11 OS-censored observations, and 46 PFS1 events and 6 PFS1-censored observations. Median follow-up was estimated using the reverse Kaplan–Meier method, in which deaths were treated as censored observations and patients censored for OS were treated as follow-up events. Median OS and median PFS1 were estimated using the Kaplan–Meier product-limit method. Confidence intervals for median survival times were obtained by inversion of the pointwise complementary log–log-transformed Kaplan–Meier confidence bands. Time-specific OS and PFS1 probabilities and their 95% confidence intervals were estimated using Greenwood variance with complementary log–log transformation. Estimates are presented as median months (95% CI) or percentages (95% CI), as appropriate. NR indicates that the Kaplan–Meier estimate or the relevant confidence bound did not cross 0.50 during the observed follow-up; NE indicates that the requested time point exceeded the maximum observed follow-up in the relevant

cohort. OS was measured from initiation of first-line systemic therapy to death from any cause. PFS1 was measured from initiation of first-line systemic therapy to radiographic or clinical progression or death from any cause, whichever occurred first. The numbers of patients remaining at risk at 12 months for PFS1 were 12, 3, and 9 in the overall, chemotherapy-alone, and chemotherapy-plus-atezolizumab cohorts, respectively. The corresponding numbers at risk at 18 months for OS were 13, 5, and 8, and those at 24 months were 5, 1, and 4. Accordingly, late time-point estimates, particularly the 12-month PFS1 and 24-month OS estimates in the chemotherapy-alone group, should be interpreted cautiously. In addition, the reverse Kaplan–Meier estimate for the chemotherapy-alone cohort was based on only 2 OS-censored observations and should therefore be interpreted with caution. Treatment-group survival estimates were unadjusted and descriptive. Because first-line atezolizumab exposure was not randomly assigned, apparent between-group differences may reflect calendar time, treatment selection, baseline clinical characteristics, residual confounding, and differential follow-up maturity and should not be interpreted as causal estimates of atezolizumab efficacy. No formal between-group hypothesis test was performed in this descriptive table.

Supplementary Figure S1. Distributions of EASIX and IPI before and after log_2 transformation

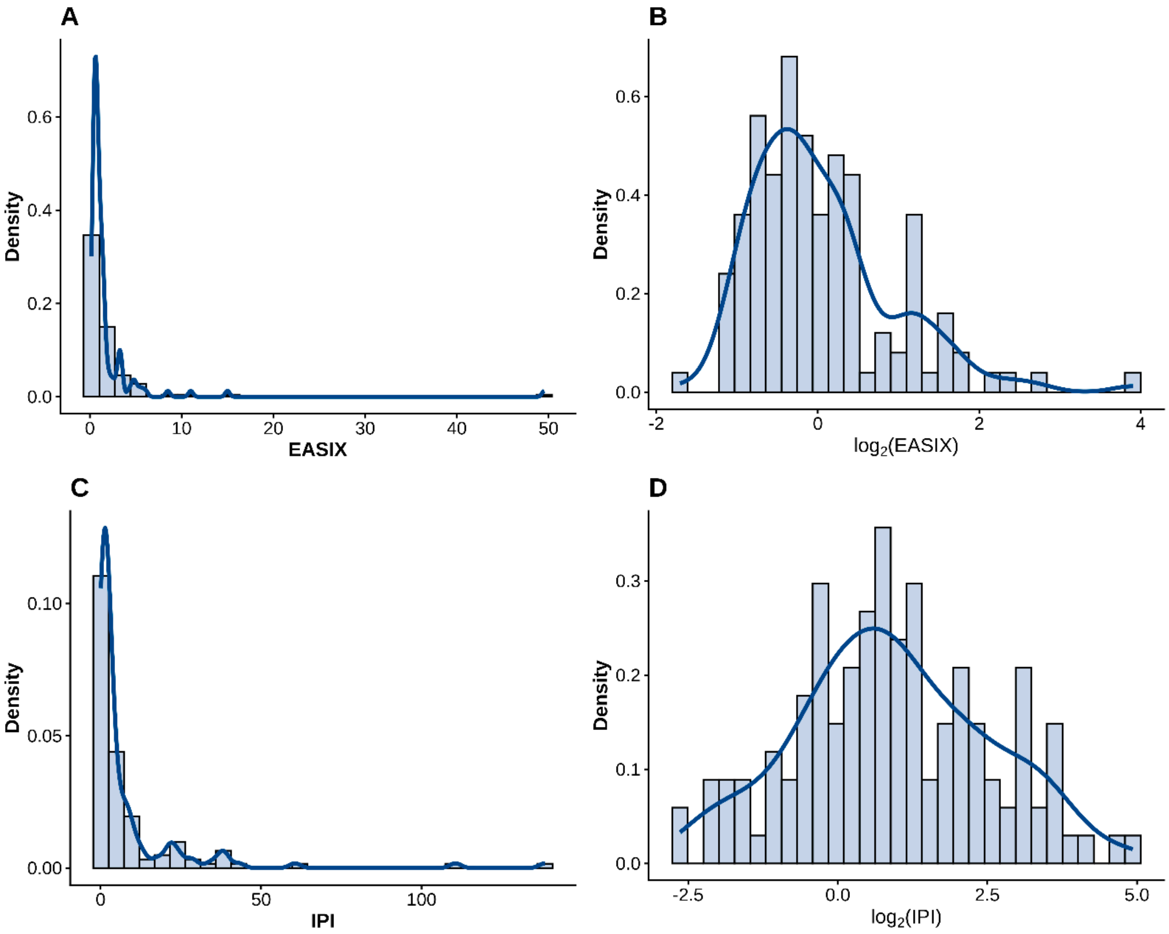

Panel A shows the distribution of the original Endothelial Activation and Stress Index (EASIX), and Panel B shows the corresponding distribution after log_2 transformation. Panel C shows the distribution of the original Inflammatory Prognostic Index (IPI), and Panel D shows the corresponding distribution after log_2 transformation. Histograms represent the observed density distributions among the 129 patients, and the superimposed solid curves represent kernel density estimates. EASIX was calculated as lactate dehydrogenase multiplied by serum creatinine and divided by platelet count. IPI was calculated as C-reactive protein multiplied by the neutrophil-to-lymphocyte ratio and divided by serum albumin; the neutrophil-to-lymphocyte ratio was calculated as the absolute neutrophil count divided by the absolute lymphocyte count. Log_2 transformation was applied to reduce right skewness, improve the distributional characteristics of the indices, and allow Cox regression hazard ratios to be interpreted as the relative change in hazard associated with each doubling of the respective index. Complete EASIX and IPI data were available for all 129 patients.

Abbreviations: CRP, C-reactive protein; EASIX, Endothelial Activation and Stress Index; IPI, Inflammatory Prognostic Index; LDH, lactate dehydrogenase; NLR, neutrophil-to-lymphocyte ratio.

Supplementary Figure S2. Spearman correlation matrix of EASIX, IPI, and selected baseline clinical and laboratory variables

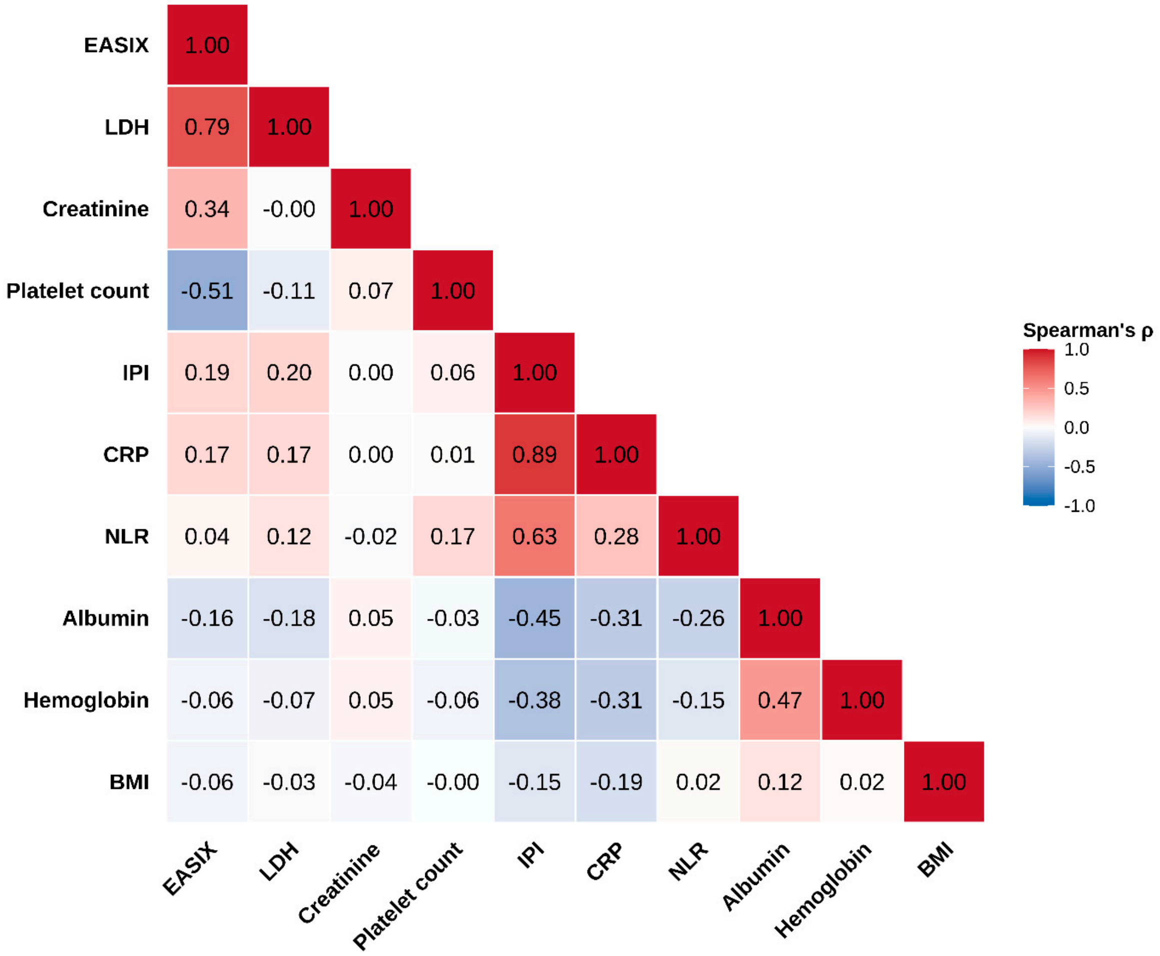

Footnote: The heatmap shows pairwise Spearman rank-correlation coefficients among the Endothelial Activation and Stress Index (EASIX), Inflammatory Prognostic Index (IPI), their constituent laboratory variables, hemoglobin, and body mass index in the complete cohort of 129 patients. Variables were ordered to group each composite index with its corresponding laboratory components. Cells display Spearman's ρ values rounded to two decimal places, with the color scale ranging from −1 to +1 and centered at 0. Only the lower triangular portion of the symmetric correlation matrix is displayed to avoid duplication. EASIX was calculated as lactate dehydrogenase multiplied by serum creatinine and divided by platelet count. IPI was calculated as C-reactive protein multiplied by the neutrophil-to-lymphocyte ratio and divided by serum albumin; the neutrophil-to-lymphocyte ratio was calculated as the absolute neutrophil count divided by the absolute lymphocyte count. Because Spearman correlation is invariant to monotonic transformation, identical coefficients would be obtained using $\log_2$-transformed EASIX and IPI. Correlations between each composite index and its constituent variables should be interpreted as partly structural because these variables are incorporated directly into the corresponding score formula. The matrix was intended as a descriptive assessment of the magnitude and direction of pairwise associations and should not be interpreted as evidence of causal or independent relationships. *P* values and significance symbols were not displayed because no inferential conclusions were based on individual pairwise correlation tests.

Abbreviations: BMI, body mass index; CRP, C-reactive protein; EASIX, Endothelial Activation and Stress Index; IPI, Inflammatory Prognostic Index; LDH, lactate dehydrogenase; NLR, neutrophil-to-lymphocyte ratio; ρ, Spearman rank-correlation coefficient.
